# Development and validation of a predictive model for depression risk in the U.S. adult population: Evidence from the 2007–2014 NHANES

**DOI:** 10.1186/s40359-023-01278-0

**Published:** 2023-08-25

**Authors:** Wei Tian, Yafeng Zhang, Xinhao Han, Yan LI, Junping Liu, Hongying Wang, Qiuju Zhang, Yujie Ma, Guangcan Yan

**Affiliations:** 1 Department of Biostatistics, School of Public Health, Harbin Medical University, Harbin, China; 2 Department of Cell Biology, Harbin Medical University, Harbin, China; 3 Institute for Hospital Management of Henan Province, The First Affiliated Hospital of Zhengzhou University, Zhengzhou, China; 4 Department of Social Medicine, School of Health Management, Harbin Medical University, Harbin, China

**Keywords:** Physical activity, Blood lead, Depression, Predictive model

## Abstract

**Background:**

Depression is a prevalent mental health disorder with a complex etiology and substantial public health implications. Early identification of individuals at risk for depression is crucial for effective intervention and prevention efforts. This study aimed to develop a predictive model for depression by integrating demographic factors (age, race, marital status, income), lifestyle factors (sleep duration, physical activity), and physiological measures (hypertension, blood lead levels). A key objective was to explore the role of physical activity and blood lead levels as predictors of current depression risk.

**Methods:**

Data were extracted from the 2007–2014 National Health and Nutrition Examination Survey (NHANES). We applied a logistic regression analysis to these data to assess the predictive value of the above eight factors for depression to create the predictive model.

**Results:**

The predictive model had bootstrap-corrected c-indexes of 0.68 (95% CI, 0.67–0.70) and 0.66 (95% CI, 0.64–0.68) for the training and validation cohorts, respectively, and well-calibrated curves. As the risk of depression increased, the proportion of participants with 1.76 ~ 68.90 µg/L blood lead gradually increased, and the proportion of participants with 0.05 ~ 0.66 µg/L blood lead gradually decreased. In addition, the proportion of sedentary participants increased as the risk of depression increased.

**Conclusions:**

This study developed a depression risk assessment model that incorporates physical activity and blood lead factors. This model is a promising tool for screening, assessing, and treating depression in the general population. However, because the corrected c-indices of the predictive model have not yet reached an acceptable threshold of 0.70, caution should be exercised when drawing conclusions. Further research is required to improve the performance of this model.

**Supplementary Information:**

The online version contains supplementary material available at 10.1186/s40359-023-01278-0.

## Introduction

Depression is one of the most common chronic diseases in adulthood and a major human blight [1, 2]. According to the World Health Organization (WHO), approximately 350 million people worldwide suffer from depression [3]. In addition, multiple prospective longitudinal studies suggest that the lifetime prevalence of depression ranges from 30–50% [4–6]. Depression is inherently unpredictable, and the duration, number, and features of depressive episodes vary [7]. Although there are scales to screen for depression, it remains difficult to define the current risk of depression. For example, while the Patient Health Questionnaire-9 (PHQ-9) is a highly useful screening tool, it is not a standalone diagnostic test [22]. It is necessary to create accurate assessments to screen for the current risk of depression to determine best practices for prevention and treatment options.

Sociodemographic factors, such as gender, age, marital status, race, and income, are associated with depression [8–10]. In addition, short and long sleep durations are significantly associated with an increased risk of depression, particularly insomnia, which is both a common symptom of depression and a risk factor for depressive episodes [11, 12]. Generally, women tend to be more susceptible to depression than men [13–15], but among people with hypertension, men had a slightly greater risk of depression than women [16]. Moreover, people with hypertension experience more prevalent sleep disturbances, which lead to more depression-mediated sleep disorders [17]. Converging evidence has suggested that changes in certain biological indicators are associated with depression [18]. Lead (Pb) is a known neurotoxicant that can cause depression by affecting brain-derived neurotrophic factors and the hypothalamic–pituitary–adrenal (HPA) axis [19, 20]. Physical activity (PA) also significantly contributes to depression. Specifically, decreased PA and increased sedentary behaviors increase the risk of depression; meanwhile, PA has antidepressant effects and can be used in addition to pharmacotherapy and psychotherapy to treat depression [21, 22]. Exercise can also have a positive effect on health behaviors associated with depression, such as sleep time [23, 24]. Although demographic factors, sleep duration, hypertension, lead intake, and PA are associated with depression, information on how these factors are linearly combined to explain the current individual risk of depression is scattered and remains unclear. In response to this gap in the literature, this study used demographic and depression-related risk factors to develop a risk predictive model for depression. Specifically, the study sought examined the predictive effects of age, race, marital status, income, sleep duration, hypertension, blood lead (PbB), and PA on depression to develop and validate this predictive model. Notably, we used the PHQ-9 to build our predictive model; therefore, our model will allow clinicians to further determine the severity of depression using the current gold standard. In doing so, the study contributes to the identification of target populations at risk of depression and timely treatment strategies for depression.

## Methods

### Study design and participants

This study used data from the National Health and Nutrition Examination Survey (NHANES) 2007–2014, an ongoing observational study designed to assess the prevalence of major diseases and risk factors for diseases in U.S. adults [25].

We downloaded the NHANES data in August 2020. The NHANES draws a representative sample of approximately 5,000 non-institutionalized civilians across the country every two year and collects information on demographics, sleep duration, hypertension, PA, PbB, and depression from 20-year-old participants. We cleaned and merged the data between September and October 2020. We analyzed the data and developed the predictive model from November to December 2020. The inclusion and exclusion criteria are shown in Additional file 1: Figure S1. Sample participants with missing information in the merged data were excluded.. It is worth noting that although the NHANES 2007–2014 includes variables associated with depression, such as smoking, alcohol consumption, BMI, and diabetes, we excluded these factors due to missing values and used demographic factors, sleep duration, hypertension, PA, and PbB as candidate predictors for the predictive model. A comparison of the baseline information of participants with missing and complete data in the training and validation cohorts is shown in Additional file 1: Tables S1-S2. The NHANES is approved by the local institutional review board [26] and participants provide written informed consent. See the NHANES website (www.cdc.gov/nchs/nhanes.htm) for further details.

## Measurements

### Depression

This study used the Patient Health Questionnaire-9 (PHQ-9) to screen for depression. The PHQ-9 is a 9-item self-report questionnaire. It is the most commonly used screening tool for depression in primary care (although it is frequently used with a tenth item: the extent to which these questions currently cause difficulty) [27]. Respondents are asked to rate each item on a scale of 0 to 3 based on how much a symptom bothered them over the last two weeks (0 = not at all, 1 = several days, 2 = more than half the days, 3 = nearly every day). The standard cut-off score for screening to identify possible depression is generally 10 or above; however, studies suggest that the probability of depression should be estimated using the full spectrum of PHQ-9 screening scores. We used an algorithm to define depression based on the DSM-IV criteria and cut-off summative item scores [28]. The algorithm requires at least five symptoms to be rated at at least 2 (more than half a day), with the exception of the suicidal ideation item, which counts as one of the five symptoms if rated at at least 1 (several days). The algorithm also requires that at least one of the symptoms scored at at least 2 be a loss of interest, pleasure, or depressed mood. Alternatively, a cutoff score of 10 or above on the summed item score is diagnosed as depression. Finally, the tenth item is added to the diagnostic part of the PHQ-9 to measure how difficult the problems identified make it for the respondent to manage work, daily living, and relationships.

### Physical activity

PA was measured using the Physical Activity Questionnaire, which includes questions related to daily, leisure-time, and sedentary activities. Here, we only considered recreational and sedentary activities. The suggested metabolic equivalent (MET) scores of activities according to the 2008 Physical Activity Guidelines for Americans classify recreational activity into four levels: sedentary (0–39 MET-minutes per week), inadequate leisure-time PA (less than 500 MET–minutes per week), moderate leisure-time PA500–1000 MET-minutes per week), and vigorous leisure-time PA (more than 1000 MET–minutes per week) [29].

### Blood lead

Medical laboratory technicians measured PbB at a mobile examination center. Non-fasting blood samples (minimum 0.25 ml/vial) were collected by venipuncture, and PbB concentrations were determined using inductively coupled plasma mass spectrometry. Detailed instructions for specimen collection and processing are publicly available on the NHANES websites. The limit of detection was 0.05 µg/L for NHANES 2007–2014. In practice, PbB is extremely skewed. In data processing, researchers typically convert extremely biased data into ordered categorical variables. Considering the predictive effect of the variables, we used the quartile values of the PbB variable, namely grades 1, 2, 3, and 4. Overall, PbB levels in the four groups ranged from 0.05 ~ 0.66, 0.66 ~ 1.07, 1.07 ~ 1.76 and 1.76 ~ 68.90µg/L respectively.

### Other variables

Several demographic factors were included in this analysis. Specifically, we collected information on participant age, gender, marital status, race, and monthly household income (< = 4: USD 0–1649, <  = 8: USD 1650–4599, and <  = 12: USD 4600 and over). It is worth noting that the monthly household income variable was also considered skewed; accordingly, we transformed and used its tertiles. Sleep duration was assessed using the National Health and NHANES Sleep Disorders Questionnaire. During the interview, participants advised on when they fell asleep during the main sleep period, with values entered directly for sleep durations of 1–11 h and “12 or more hours” used for sleep durations of over 12 h. Similar to another study, a “short” sleep duration was identified as < 6 h per day, a “normal” sleep duration as 6–8 h per day, and a “long” sleep duration as > 8 h per day in the present study [30]. Hypertension was defined as an average systolic blood pressure of ≥ 140 mmHg, diastolic blood pressure of ≥ 90 mmHg, or treatment with hypertensive medications [31].

### Statistical analyses

First, the two cohorts were statistically described, and the distribution characteristics of PbB and PA were compared between participants with and without depression. Continuous variables were compared using an unpaired, 2-tailed t-test or Mann–Whitney tests, while categorical variables were compared using χ^2^ tests. Second, a logistic regression analysis was performed with depression as the dependent variable and demographics, sleep duration, hypertension, PA, and PbB as the independent variables, and a predictive model was developed.

We developed a nomogram based on the results of the model and validated it internally and externally (using the bootstrapping method). The nomogram, which is developed using a logistic regression model, is a useful tool for predicting risk by combining multiple predictors and visualizing the probability of the outcome. In particular, of all available models, a nomogram can provide individualized, evidence-based, and highly accurate risk estimation [23]; notably, it has the advantage of visualizing complex statistical predictive models as risk estimates of individualized disease probabilities [24, 25]. To develop a nomogram, a logistic regression model was constructed using the rms R package, and the nomogram function was used to visualize the regression results. The nomogram was based on proportionally converting each regression coefficient in the multivariate logistic regression to a 0–100-point scale. The variable with the highest β coefficient (absolute value) was assigned 100 points. Points were added across independent variables to derive total points, which were converted to predicted probabilities. Specifically, the predictive factors were plotted on an axis and the risk contributions of each predictive factor were used to calculate the predicted probabilities for the study subjects. The predictive factor with the highest OR value was assigned a score of 100 for all predictive factors, whereas the other influencing factors were assigned proportional scores. Finally, the patients’ total risk score was calculated by drawing a straight line from the point corresponding to each predictive factor on the vertical axis. From the graph, the predicted probability was read using the probability of the results marked on the vertical axis. Calibration curves and a corrected Harrell C-index were used to measure the predictive performance of the nomograms.

Third, given the possible impact of the sampling weights of the NHANES on the predictive model, 1000 cohort with the same number of training cohort were generated by weighted random sampling, and the area under the receiver operating characteristic curve (AUC) value of the predictive model constructed by each cohort was calculated separately to evaluate the stability of the model. Fourth, compared to the model without PA and PbB, net reclassification improvement (NRI) and integrated discrimination improvement (IDI) were used to evaluate improvements in the nomogram. Stratified analysis was performed to determine the association between PA, PbB, and the risk of depression. A network calculator based on a predictive model was developed to provide a convenient, fast, and intuitive tool for individualized forecasting. All analyses were performed using R version (version 3.3.2) (http://www.r-project.org/), and *P* < 0.05 was considered statistically significant.

## Results

From the participants enrolled in the NHANES 2007–2014, we identified 9971 subjects who met the inclusion criteria, of whom 5956 were assigned to the training cohort (NHANES 2007–2010) and 4015 to the validation cohort (NHANES 2007–2014). Descriptive statistics of the demographic characteristics and risk factors of the study populations in the training and validation cohorts are provided in Additional file 1: Table S3. The baseline characteristics of the study population with depression among the training and validation cohorts are listed in Tables 1 and S4, respectively. All baseline data were significantly different between the depression and non-depression groups in both cohorts (*P* < 0.05). Depression was found in 1154 (19.4%) and 855 (21.3%) patients in the two cohorts, respectively. Compared to participants without depression, participants with depression in the two cohorts had higher rates of sedentary time and lower leisure-time PA (*P* < 0.05). In the training cohort, the prevalence of depression was 8.3%, 25.7%, 29.0%, and 36.9% in the four PbB quantiles, respectively. In the validation cohort, the prevalence of depression was 19.3%, 23.9%, 28.7%, and 28.2% in the 4 four PbB quantiles, respectively.

**Table 1:** Descriptive statistics of the study population in depression among the training cohort

Factors	Levels	Overall (*n* = 5956), %	Depression (*n* = 1154), %	Non-depression (*n* = 4802), %	*P* value
Gender	Male	2692 (45.2)	477 (41.3)	2215 (46.1)	0.004
	Female	3264 (54.8)	677 (58.7)	2587 (53.9)	
Age	20 ~ 29	1041 (17.5)	152 (13.2)	889 (18.5)	< 0.001
	30 ~ 39	1013 (17.0)	178 (15.4)	835 (17.4)	
	40 ~ 49	1100 (18.5)	236 (20.5)	864 (18.0)	
	50 ~ 59	947 (15.9)	222 (19.2)	725 (15.1)	
	60 ~ 69	908 (15.2)	194 (16.8)	714 (14.9)	
	> = 70	947 (15.9)	172 (14.9)	775 (16.1)	
Race	Hispanic	988 (16.6)	230 (19.9)	758 (15.8)	< 0.001
	Non-Hispanic White	3127 (52.5)	520 (45.1)	2607 (54.3)	
	African American	1024 (17.2)	231 (20.0)	793 (16.5)	
	Other	817 (13.7)	173 (15.0)	644 (13.4)	
Marry	Married/Cohabitting	3469 (58.2)	572 (49.6)	2897 (60.3)	< 0.001
	Unmarried	1056 (17.7)	213 (18.5)	843 (17.6)	
	Wid/Div/Sep	1431 (24.0)	369 (32.0)	1062 (22.1)	
Physical activity	Vigorous	1122 (18.8)	125 (10.8)	997 (20.8)	< 0.001
	Inadequate	812 (13.6)	113 (9.8)	699 (14.6)	
	Moderate	627 (10.5)	66 (5.7)	561 (11.7)	
	Sedentary	3395 (57.0)	850 (73.7)	2545 (53.0)	
Income	< = 4	2048 (34.4)	566 (49.0)	1482 (30.9)	< 0.001
	< = 8	2382 (40.0)	433 (37.5)	1949 (40.6)	
	< = 12	1526 (25.6)	155 (13.4)	1371 (28.6)	
Sleep time	< 6 h	1126 (18.9)	321 (27.8)	805 (16.8)	< 0.001
	< = 8 h	4394 (73.8)	713 (61.8)	3681 (76.7)	
	> 8 h	436 (7.3)	120 (10.4)	316 (6.6)	
Hypertension	Yes	2545 (42.7)	590 (51.1)	1955 (40.7)	< 0.001
	No	3411 (57.3)	564 (48.9)	2847 (59.3)	
Blood lead	Grade 1	754 (12.7)	96 (8.3)	658 (13.7)	< 0.001
	Grade 2	1447 (24.3)	297 (25.7)	1150 (23.9)	
	Grade 3	1779 (29.9)	335 (29.0)	1444 (30.1)	
	Grade 4	1976 (33.2)	426 (36.9)	1550 (32.3)	

*Abbreviations: NHANES *National Health and Nutrition Examination Survey, *Wid *Widowed, *Div *Divorced, *Sep *Separated, Sleep time was categorized as < 6 h, 6 - 8 h and > 8 h. Monthly household income included lower income (< = 4: $0-$1649), moderate income (< = 8: $1650-$4599) and high income (< = 12: $4600 and over). Blood lead were analyzed by dividing them into four quintiles according to the range of values

**Fig. 1:**
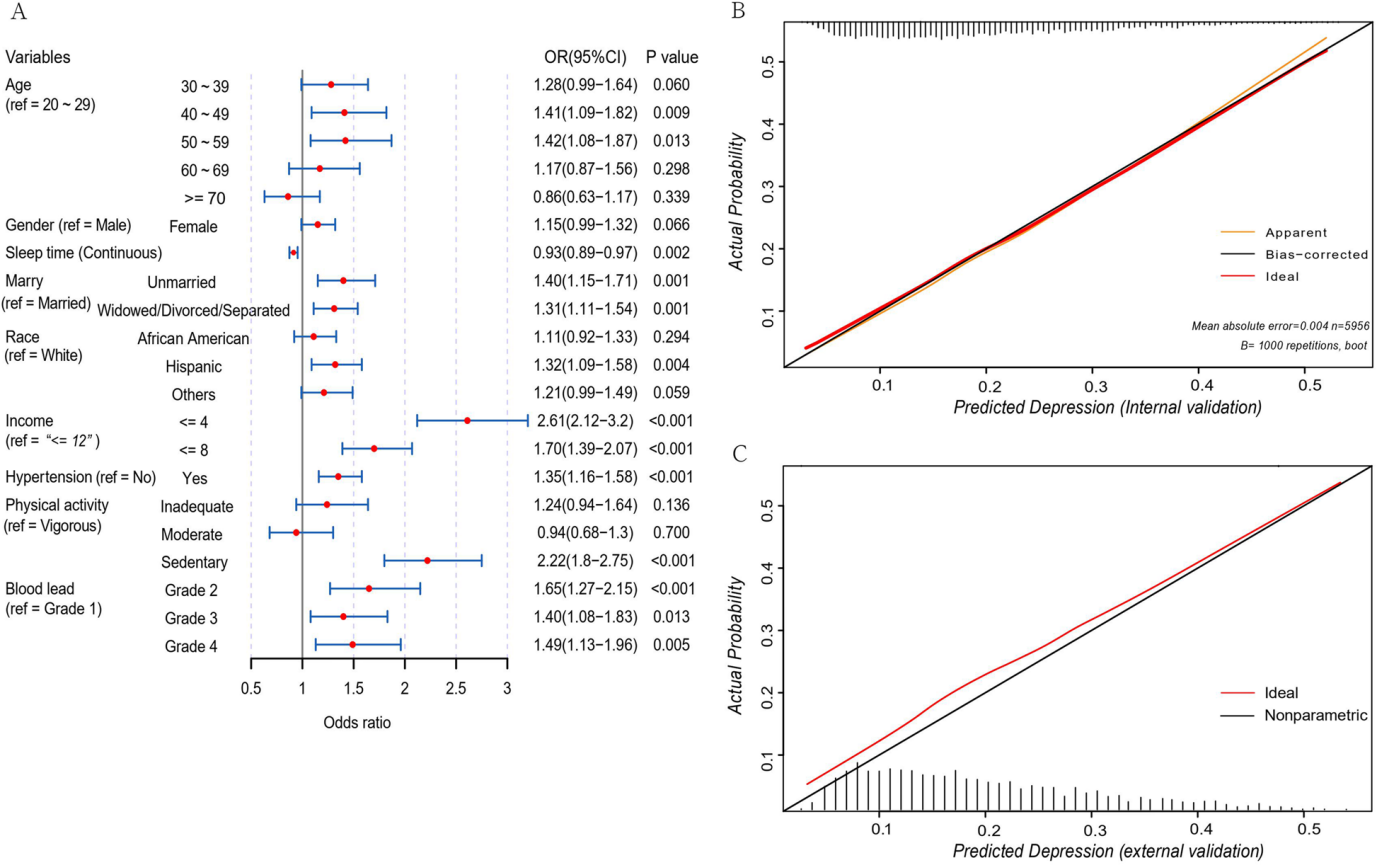
Odds ratio (95% CI) values between predictors and depression and validation results of the prediction model. **A** Results of logistic regression analysis with depression as an outcome variable, (**B**) Calibration curve of the prediction nomogram in the primary cohort, (**C**) Calibration curve of the prediction nomogram in the validation cohort

The logistic regression analysis with depression as the outcome variable identified age, sleep duration, marital status, race, income, hypertension, PA, and PbB level as independent predictors (Fig. 1A). In the logistic regression analysis, the results were reported as odds ratios (95% CI). It should be noted that gender was excluded from the model because of its statistically insignificant association with depression (1.15 [0.99–1.32]). We found a greater risk of depression in participants who were sedentary than in those who engaged in vigorous leisure-time PA (2.22 [1.80–2.75]). The odds ratios for depression were 1.65 [95%CI, 1.27–2.15], 1.40 [95%CI, 1.08–1.83]. and 1.49 [95%CI, 1.13–1.96] for grade 2, grade 3, and grade 4 of PbB, respectively, in comparison with grade 1 of PbB.

**Fig. 2:**
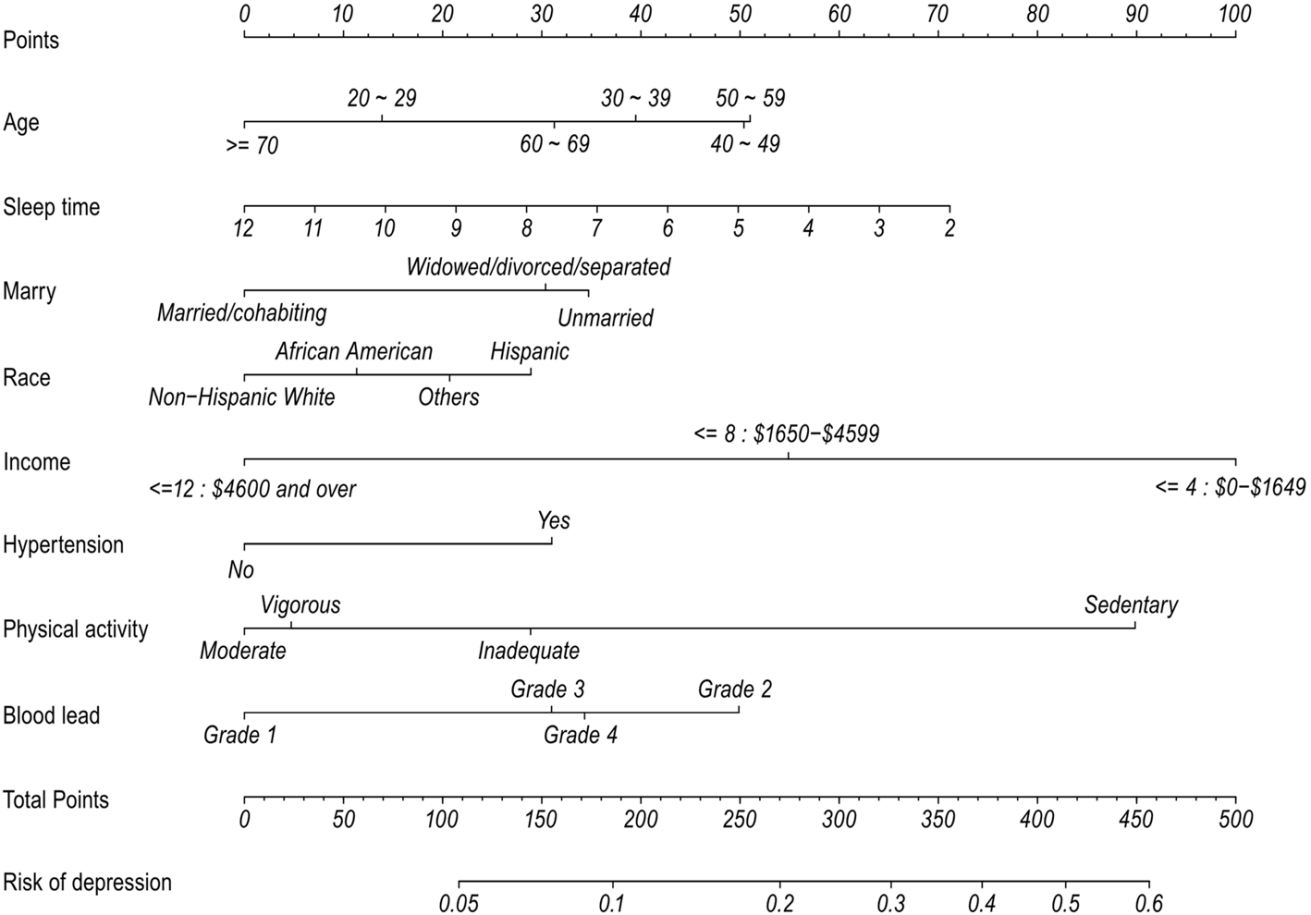
Nomogram for estimating the probability of current individualized depression risk

**Fig. 3:**
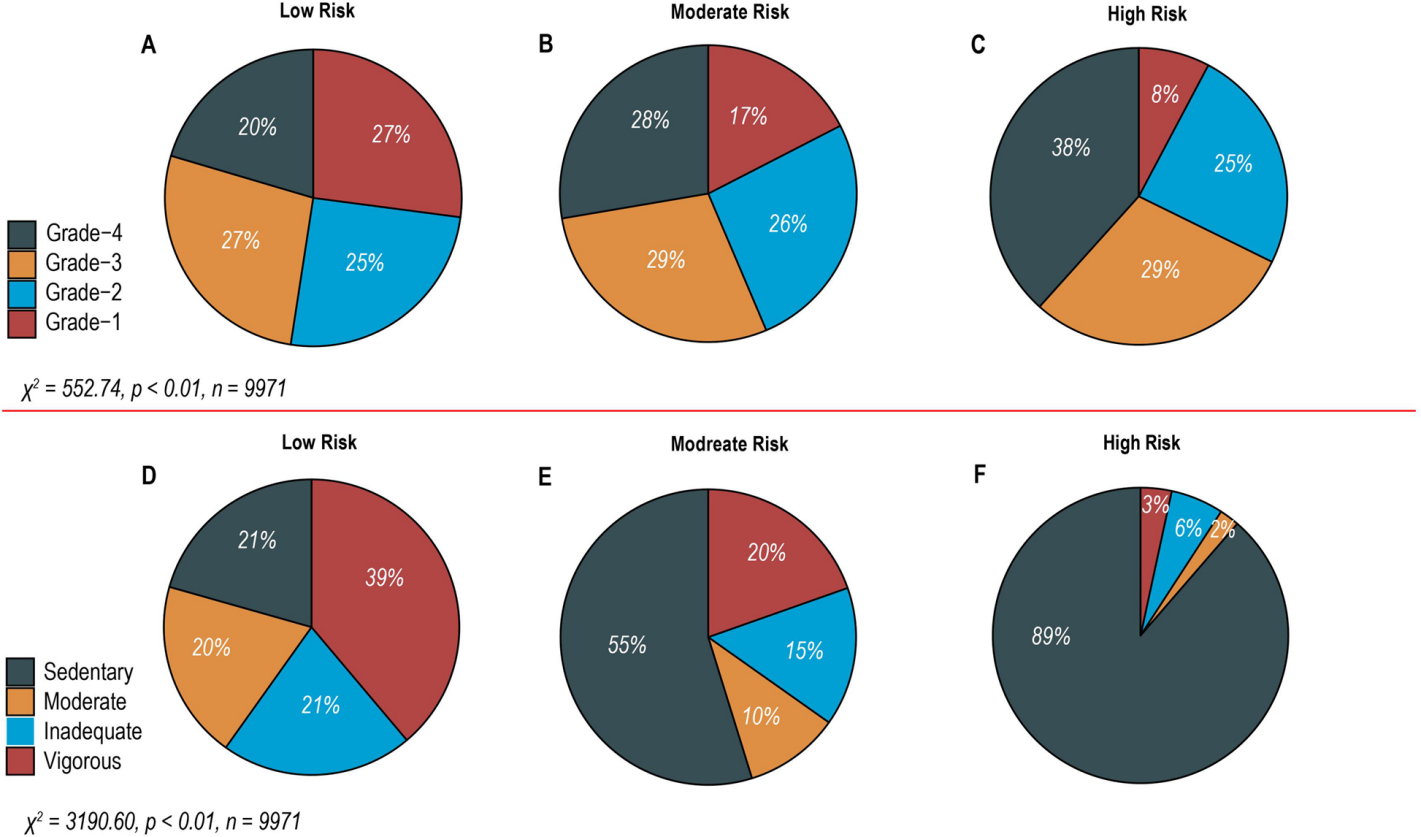
Association between blood lead, physical activity and different depression risks

These independently associated risk factors were used to develop a nomogram for estimating the probability of depression (Fig. 2). The established depression predictive model showed good discrimination with a bootstrap-corrected C-index of 0.68 (95%CI, 0.67–0.70). Similarly, the bootstrap-corrected C-index for the depression risk predictive model in the validation cohort was 0.66 (95% CI, 0.64–0.68). Furthermore, the calibration curves of the predictive model in both the training and validation cohorts agreed well with the actual and predicted probabilities of depression (Fig. 1B and C). The stability of the predictive model was further demonstrated by the mean AUC of 0.66 (95% CI, 0.63–0.69) for 1000 predictive models under 1000 weighted random samples. In addition, the net reclassification improvement (NRI) for PbB alone was 0.02 (CI:0.01–0.04), *P* < 0.05, and the NRI for PA alone was 0.09 (95% CI, 0.06–0.12), *P* < 0.05; NRI for PA on basic of PbB was 0.11 (CI:0.09–0.14), *P* < 0.05. From the integrated discrimination improvement (IDI) calculations, adding PA and PbB improved the predicted probability of the model by 0.02 (95%, 0.017–0.024; *P* < 0.001) compared to the model without PA and PbB, and PA alone improved the IDI by 0.018 (95%, 0.014–0.021; *P* < 0.001). Depression was categorized into three subgroups according to the nomogram-estimated risk of depression (across three quantiles): low risk, moderate risk, and high risk. The composition of PbB and PA in each of the three depression risk subgroups is presented in Fig. 3. Across the three quantiles, the proportion of grade 4 PbB gradually increased with an increasing risk of depression (20%, 28%, 38%), whereas grade 1 PbB gradually decreased (27%, 17%, 8%). In addition, as the risk of depression increased, there was a gradual increase in the proportion of sedentary behavior (21%, 55%, 89%) and a gradual decrease in the proportions of inadequate PA (21%, 15%, 6%), moderate PA (20%, 10%, 2%), and vigorous PA (39%, 20%, 3%).

## Discussion

In this study, we created a depression predictive model by combining demographics, sleep duration, hypertension, PA, and PbB levels to identify current risk factors for depression. The calibration and discrimination of the model were well-validated and can be generalized to the US civilian population. The inclusion of PbB and PA risk factors in the developed predictive model holds significant promise for studying the risk of depression.

Although depression risk models have been developed for cancer outpatients, these models cannot be used to screen for depression in the population due to their insufficient sample size, limited representation, and lack of external validation. However, existing knowledge offers the foundation for the creation of a large-scale screening tool. We know that low levels of PA correlate with higher levels of depression [32, 33]. In addition, scholars have established that lead is one of the most dangerous environmental toxins. Although the safe blood concentration of lead remains unknown, long-term lead exposure clearly leads to low and moderate PbB concentrations, which are significantly associated with depression [34, 35]. While human exposure to lead occurs in a variety of ways, it is primarily associated with environmental contamination. Currently, minimizing lead exposure in the workplace, home, and broader environment is an important part of public health interventions and policies. Primary strategies include nationwide lead monitoring programs (including the monitoring of PbB concentrations across populations), raising lead awareness, increasing health literacy, teaching skills to prevent lead poisoning, developing and implementing comprehensive regulatory and health intervention programs to reduce lead exposure and associated health risks, and promoting lead-focused nutritional interventions.

Our results suggest that PbB concentrations above 0.66µg/L are associated with an increased risk of depression. Therefore, considerations of the health risks of lead exposure should take depression into account. However, to the best of our knowledge, no studies have yet applied PbB and PA factors to predict the current risk of individualized depression. While our study responds to this gap, it is important to note that the discrimination of our model was only approximately 0.70; this suggests that we still need to identify more important predictors for inclusion in the model. In addition, the web calculator, designed based on the model, extends the use of the study and provides convenient screening for depression in the general population.

Evidence suggests that increased PbB levels are associated with a higher risk of depression [34, 36]. Our study also found that increases in grade 4 PbB and decreases in grade 1 PbB are associated with an increased risk of depression, suggesting that high PbB levels are a risk factor for depression—this may be due to the damage that lead can cause to the nervous system and the changes it can create in biological mechanisms involved in the pathophysiology [37, 38]. However, there is no conclusive evidence suggesting that PbB affects depression at specific thresholds. Given that it is possible to screen for and trigger depression, addressing this knowledge gap has important practical implications. We found that there were no significant differences in the relationships between changes in grade 2 and grade 3 levels of PbB and the risk of depression. Meanwhile, as discussed above, several studies have shown that PA is beneficial for a range of chronic diseases and that exercise is effective in the treatment of mild to moderate depression [39]. In contrast, the present study found that individuals with high levels of PA were less likely to develop depression than those with low levels [21]. We confirmed that a higher proportion of people with a moderate and high risk of depression were sedentary than those with a low risk. Therefore, given a moderate or high risk of depression, it is important to avoid sedentary activity and increase PA.

### Limitations

This study had a few limitations. First, while the nomogram developed in this study was highly calibrated, the discrimination was around 0.70, indicating that more sensitive and important indicators must be included in the predictive model. Second, due to missing variables, we excluded depression-related variables, such as chronic illness, from the predictor selection; this could have caused selective bias in the models. Finally, the PHQ-9 scale applied in this study cannot be used to diagnose depression; therefore, the risk of depression assessed using the predictive model needs to be further validated using the gold standard.

In conclusion, this study developed a predictive model for identifying the risk of depression using demographic characteristics, sleep duration, hypertension, PbB levels, and PA. Based on our findings, we created an individualized prediction web calculator that can be used to easily, quickly, and accurately predict the current individual depression risk and dynamic process of prediction probability at https://hmuhan157-account.shinyapps.io/Web-calculator/. Web calculators contribute significantly to public health because they enable individuals to self-assess their current risk of depression, change their negative mood in a timely manner, and work on their positive psychological development. More research is needed to further enhance the effectiveness of our predictive model for depression risk, to determine the dose–response relationship between PbB level and depression risk, and to clarify the underlying mechanism of the influence of PA on depression risk.

### Supplementary Information

**Additional file 1:** **Table S1.** Comparison of baseline information for participants with missing data and complete data in the training cohort. **Table S2.** Comparison of baseline information for participants with missing data and complete data in the validation cohort. **Table S3.** Descriptive statistics of the study population among the training cohort and validation cohort, NHANES, 2007–2014. **Table S4.** Descriptive statistics of the study population in depression among the validation cohort, NHANES, 2011–2014. **Fig. S1.** Samples flow chart.

## Data Availability

The datasets generated and/or analysed during the current study are available in the [National Health and Nutrition Examination Survey] repository, [https://wwwn.cdc.gov/Nchs/Nhanes/continuousnhanes/default.aspx].

## Abbreviations

NHANES: National Health and Nutrition Examination Survey
WHO: World Health Organization
HPA: Hypothalamic–pituitary–adrenal
PA: Physical activity
PHQ-9: Patient Health Questionnaire-9
MET: Metabolic equivalent
AUC: Area under the receiver operating characteristic curve
NRI: Net reclassification improvement
IDI: Integrated discrimination improvement
