# Supplementary material for: Development and validation of a predictive model for depression risk in the U.S. adult population: Evidence from the 2007–2014 NHANES
Source: BMC Psychol. 2023 Aug 25;11:244. doi: 10.1186/s40359-023-01278-0 (PMC10463541; doi:10.1186/s40359-023-01278-0)
Supplement: Supplementary file 1 — Additional file 1: Table S1. Comparison of baseline information for participants with missing data and complete data in the training cohort. Table S2. Comparison of baseline information for participants with missing data and complete data in the validation cohort. Table S3. Descriptive statistics of the study population among the training cohort and validation cohort, NHANES, 2007–2014. Table S4. Descriptive statistics of the study population in depression among the validation cohort, NHANES, 2011–2014. Fig. S1. Samples flow chart. [file 40359_2023_1278_MOESM1_ESM.docx]

Table S1. Comparison of baseline information for participants with missing data and complete data in the training cohort.

|  | level | Overall  (n = 18109),% | Missing data  (n = 12153),% | Complete data  (n = 5956),% | P value |
| --- | --- | --- | --- | --- | --- |
| Age | 20 ~ 29 | 2980 (16.5) | 1939 (16.0) | 1041 (17.5) | <0.001 |
|  | 30 ~ 39 | 3067 (16.9) | 2054 (16.9) | 1013 (17.0) |  |
|  | 40 ~ 49 | 3157 (17.4) | 2057 (16.9) | 1100 (18.5) |  |
|  | 50 ~ 59 | 2823 (15.6) | 1876 (15.4) | 947 (15.9) |  |
|  | 60 ~ 69 | 2868 (15.8) | 1960 (16.1) | 908 (15.2) |  |
|  | >= 70 | 3214 (17.7) | 2267 (18.7) | 947 (15.9) |  |
| Income | <= 12 | 4285 (25.9) | 2759 (26.1) | 1526 (25.6) | 0.194 |
|  | <= 4 | 5534 (33.5) | 3486 (33.0) | 2048 (34.4) |  |
|  | <= 8 | 6700 (40.6) | 4318 (40.9) | 2382 (40.0) |  |
| Race | African American | 3373 (18.6) | 2349 (19.3) | 1024 (17.2) | <0.001 |
|  | Hispanic | 1891 (10.4) | 1298 (10.7) | 593 (10.0) |  |
|  | Others | 3981 (22.0) | 2769 (22.8) | 1212 (20.3) |  |
|  | White | 8864 (48.9) | 5737 (47.2) | 3127 (52.5) |  |
| Gender | Male | 8608 (47.5) | 5916 (48.7) | 2692 (45.2) | <0.001 |
|  | Female | 9501 (52.5) | 6237 (51.3) | 3264 (54.8) |  |
| Sleep time | 6 ~ 9 hours | 14508 (80.2) | 9830 (81.1) | 4678 (78.5) | <0.001 |
|  | < 6 hours | 3075 (17.0) | 1949 (16.1) | 1126 (18.9) |  |
|  | > 9 hours | 497 (2.7) | 345 (2.8) | 152 (2.6) |  |
| Marry | MC | 10655 (58.9) | 7186 (59.2) | 3469 (58.2) | 0.479 |
|  | Unmarried | 3147 (17.4) | 2091 (17.2) | 1056 (17.7) |  |
|  | WDS | 4299 (23.8) | 2868 (23.6) | 1431 (24.0) |  |
| Physical activity | Vigorous | 3560 (19.7) | 2438 (20.1) | 1122 (18.8) | 0.216 |
|  | Inadequate | 2400 (13.3) | 1588 (13.1) | 812 (13.6) |  |
|  | Moderate | 1869 (10.3) | 1242 (10.2) | 627 (10.5) |  |
|  | Sedentary | 10280 (56.8) | 6885 (56.7) | 3395 (57.0) |  |
| Hypertension | No | 9833 (56.2) | 6422 (55.7) | 3411 (57.3) | 0.053 |
|  | Yes | 7648 (43.8) | 5103 (44.3) | 2545 (42.7) |  |
| Blood lead | Grade 1 | 2034 (11.9) | 1280 (11.5) | 754 (12.7) | 0.010 |
|  | Grade 2 | 4014 (23.5) | 2567 (23.1) | 1447 (24.3) |  |
|  | Grade 3 | 5151 (30.1) | 3372 (30.3) | 1779 (29.9) |  |
|  | Grade 4 | 5886 (34.5) | 3910 (35.1) | 1976 (33.2) |  |

MC: Married/cohabiting; WDS: Widowed/divorced/separated

Table S2. Comparison of baseline information for participants with missing data and complete data in the validation cohort.

|  | level | Overall  (n = 15344),% | Missing data  (n = 11329),% | Complete data  (n = 4015),% | P value |
| --- | --- | --- | --- | --- | --- |
| Age | 20 ~ 29 | 2713 (17.7) | 1950 (17.2) | 763 (19.0) | 0.063 |
|  | 30 ~ 39 | 2669 (17.4) | 1961 (17.3) | 708 (17.6) |  |
|  | 40 ~ 49 | 2606 (17.0) | 1934 (17.1) | 672 (16.7) |  |
|  | 50 ~ 59 | 2477 (16.1) | 1852 (16.3) | 625 (15.6) |  |
|  | 60 ~ 69 | 2516 (16.4) | 1848 (16.3) | 668 (16.6) |  |
|  | >= 70 | 2363 (15.4) | 1784 (15.7) | 579 (14.4) |  |
| Income | <= 12 | 4123 (29.4) | 3020 (30.2) | 1103 (27.5) | 0.001 |
|  | <= 4 | 4391 (31.4) | 3059 (30.6) | 1332 (33.2) |  |
|  | <= 8 | 5488 (39.2) | 3908 (39.1) | 1580 (39.4) |  |
| Race | African American | 3547 (23.1) | 2632 (23.2) | 915 (22.8) | <0.001 |
|  | Hispanic | 1480 (9.6) | 1086 (9.6) | 394 (9.8) |  |
|  | Others | 4048 (26.4) | 3098 (27.3) | 950 (23.7) |  |
|  | White | 6269 (40.9) | 4513 (39.8) | 1756 (43.7) |  |
| Gender | Male | 7312 (47.7) | 5498 (48.5) | 1814 (45.2) | <0.001 |
|  | Female | 8032 (52.3) | 5831 (51.5) | 2201 (54.8) |  |
| Sleep time | 6 ~ 9 hours | 12435 (81.2) | 9264 (81.9) | 3171 (79.0) | <0.001 |
|  | < 6 hours | 2439 (15.9) | 1703 (15.1) | 736 (18.3) |  |
|  | > 9 hours | 447 (2.9) | 339 (3.0) | 108 (2.7) |  |
| Marry | MC | 8679 (56.6) | 6505 (57.5) | 2174 (54.1) | 0.001 |
|  | Unmarried | 3191 (20.8) | 2300 (20.3) | 891 (22.2) |  |
|  | WDS | 3464 (22.6) | 2514 (22.2) | 950 (23.7) |  |
| Physical activity | sufficient | 3613 (23.5) | 2697 (23.8) | 916 (22.8) | 0.564 |
|  | insufficent | 2189 (14.3) | 1614 (14.2) | 575 (14.3) |  |
|  | moderate | 1675 (10.9) | 1242 (11.0) | 433 (10.8) |  |
|  | sede | 7866 (51.3) | 5775 (51.0) | 2091 (52.1) |  |
| Hypertension | No | 8220 (55.6) | 5994 (55.7) | 2226 (55.4) | 0.788 |
|  | Yes | 6555 (44.4) | 4766 (44.3) | 1789 (44.6) |  |
| Blood lead | Grade-1 | 2702 (23.0) | 1728 (22.4) | 974 (24.3) | 0.057 |
|  | Grade-2 | 3132 (26.7) | 2053 (26.6) | 1079 (26.9) |  |
|  | Grade-3 | 3126 (26.6) | 2071 (26.8) | 1055 (26.3) |  |
|  | Grade-4 | 2780 (23.7) | 1873 (24.2) | 907 (22.6) |  |

MC: Married/cohabiting; WDS: Widowed/divorced/separated

Table S3 Descriptive statistics of the study population among the training cohort and validation cohort, NHANES, 2007–2014.

| Factors | Levels | Overall  (n = 9971),% | Training  (n = 5956),% | | Validation  (n = 4015),% | P value |
| --- | --- | --- | --- | --- | --- | --- |
| Gender | Male | 4506 (45.2) | 2692 (45.2) | 1814 (45.2) | | 1.000 |
|  | Female | 5465 (54.8) | 3264 (54.8) | 2201 (54.8) | |  |
| Age | 20 ~ 29 | 1804 (18.1) | 1041 (17.5) | 763 (19.0) | | 0.014 |
|  | 30 ~ 39 | 1721 (17.3) | 1013 (17.0) | 708 (17.6) | |  |
|  | 40 ~ 49 | 1772 (17.8) | 1100 (18.5) | 672 (16.7) | |  |
|  | 50 ~ 59 | 1572 (15.8) | 947 (15.9) | 625 (15.6) | |  |
|  | 60 ~ 69 | 1576 (15.8) | 908 (15.2) | 668 (16.6) | |  |
|  | > = 70 | 1526 (15.3) | 947 (15.9) | 579 (14.4) | |  |
| Race | Hispanic | 1386 (13.9) | 988 (16.6) | 398 (9.9) | | <0.001 |
|  | Non-Hispanic White | 4883 (49.0) | 3127 (52.5) | 1756 (43.7) | |  |
|  | African American | 1939 (19.4) | 1024 (17.2) | 915 (22.8) | |  |
|  | Other | 1763 (17.7) | 817 (13.7) | 946 (23.6) | |  |
| Marry | Married/Cohabitting | 5643 (56.6) | 3469 (58.2) | 2174 (54.1) | | <0.001 |
|  | Unmarried | 1947 (19.5) | 1056 (17.7) | 891 (22.2) | |  |
|  | Wid/Div/Sep | 2381 (23.9) | 1431 (24.0) | 950 (23.7) | |  |
| Physical activity | Vigorous | 2038 (20.4) | 1122 (18.8) | 916 (22.8) | | <0.001 |
|  | Inadequate | 1387 (13.9) | 812 (13.6) | 575 (14.3) | |  |
|  | Moderate | 1060 (10.6) | 627 (10.5) | 433 (10.8) | |  |
|  | Sedentary | 5486 (55.0) | 3395 (57.0) | 2091 (52.1) | |  |
| Income | <= 4 | 3380 (33.9) | 2048 (34.4) | 1332 (33.2) | | 0.111 |
|  | <= 8 | 3962 (39.7) | 2382 (40.0) | 1580 (39.4) | |  |
|  | <= 12 | 2629 (26.4) | 1526 (25.6) | 1103 (27.5) | |  |
| Sleep time | < 6 hours | 1862 (18.7) | 1126 (18.9) | 736 (18.3) | | 0.730 |
|  | <= 8 hours | 7370 (73.9) | 4394 (73.8) | 2976 (74.1) | |  |
|  | > 8 hours | 739 (7.4) | 436 (7.3) | 303 (7.5) | |  |
| Hypertension | Yes | 4334 (43.5) | 2545 (42.7) | 1789 (44.6) | | 0.074 |
|  | No | 5637 (56.5) | 3411 (57.3) | 2226 (55.4) | |  |
| Blood lead | Grade 1 | 1728 (17.3) | 754 (12.7) | 974 (24.3) | | <0.001 |
|  | Grade 2 | 2526 (25.3) | 1447 (24.3) | 1079 (26.9) | |  |
|  | Grade 3 | 2834 (28.4) | 1779 (29.9) | 1055 (26.3) | |  |
|  | Grade 4 | 2883 (28.9) | 1976 (33.2) | 907 (22.6) | |  |

Abbreviations: NHANES - National Health and Nutrition Examination Survey; Wid – Widowed; Div – Divorced; Sep – Separated; sleep.time - How much sleep do you usually get at night on weekdays or workdays ? And sleep time was categorized as < 6 hours, 6 - 8 hours and > 8 hours. Education Monthly household income standard included lower income (<= 4 : $0-$1649), mediate income (<= 8: $1650-$4599) and high income (<=12: $4600 and over). Blood lead were analyzed by dividing them into four quintiles according to the range of values.

Table S4 Descriptive statistics of the study population in depression among the validation cohort, NHANES, 2011–2014.

| Factors | Levels | Overall  (n = 4015),% | Depression (n = 855),% | Non-depression (n = 3160),% | P value |
| --- | --- | --- | --- | --- | --- |
| Gender | Male | 1814 (45.2) | 353 (41.3) | 1461 (46.2) | 0.011 |
|  | Female | 2201 (54.8) | 502 (58.7) | 1699 (53.8) |  |
| Age | 20 ~ 29 | 763 (19.0) | 122 (14.3) | 641 (20.3) | <0.001 |
|  | 30 ~ 39 | 708 (17.6) | 112 (13.1) | 596 (18.9) |  |
|  | 40 ~ 49 | 672 (16.7) | 138 (16.1) | 534 (16.9) |  |
|  | 50 ~ 59 | 625 (15.6) | 182 (21.3) | 443 (14.0) |  |
|  | 60 ~ 69 | 668 (16.6) | 172 (20.1) | 496 (15.7) |  |
|  | > = 70 | 579 (14.4) | 129 (15.1) | 450 (14.2) |  |
| Race | Hispanic | 398 (9.9) | 81 (9.5) | 317 (10.0) | <0.001 |
|  | Non-Hispanic White | 1756 (43.7) | 331 (38.7) | 1425 (45.1) |  |
|  | African American | 915 (22.8) | 233 (27.3) | 682 (21.6) |  |
|  | Other | 946 (23.6) | 210 (24.6) | 736 (23.3) |  |
| Marry | Married/Cohabitting | 2174 (54.1) | 382 (44.7) | 1792 (56.7) | <0.001 |
|  | Unmarried | 891 (22.2) | 192 (22.5) | 699 (22.1) |  |
|  | Wid/Div/Sep | 950 (23.7) | 281 (32.9) | 669 (21.2) |  |
| Physical activity | Vigorous | 916 (22.8) | 134 (15.7) | 782 (24.7) | <0.001 |
|  | Inadequate | 575 (14.3) | 98 (11.5) | 477 (15.1) |  |
|  | Moderate | 433 (10.8) | 74 (8.7) | 359 (11.4) |  |
|  | Sedentary | 2091 (52.1) | 549 (64.2) | 1542 (48.8) |  |
| Income | <= 4 | 1332 (33.2) | 414 (48.4) | 918 (29.1) | <0.001 |
|  | <= 8 | 1580 (39.4) | 327 (38.2) | 1253 (39.7) |  |
|  | <= 12 | 1103 (27.5) | 114 (13.3) | 989 (31.3) |  |
| Sleep time | < 6 hours | 736 (18.3) | 204 (23.9) | 532 (16.8) | <0.001 |
|  | <= 8 hours | 2976 (74.1) | 570 (66.7) | 2406 (76.1) |  |
|  | > 8 hours | 303 (7.5) | 81 (9.5) | 222 (7.0) |  |
| Hypertension | Yes | 1789 (44.6) | 451 (52.7) | 1338 (42.3) | <0.001 |
|  | No | 2226 (55.4) | 404 (47.3) | 1822 (57.7) |  |
| Blood lead | Grade 1 | 974 (24.3) | 165 (19.3) | 809 (25.6) | <0.001 |
|  | Grade 2 | 1079 (26.9) | 204 (23.9) | 875 (27.7) |  |
|  | Grade 3 | 1055 (26.3) | 245 (28.7) | 810 (25.6) |  |
|  | Grade 4 | 907 (22.6) | 241 (28.2) | 666 (21.1) |  |

Abbreviations: NHANES - National Health and Nutrition Examination Survey; Wid – Widowed; Div – Divorced; Sep – Separated; sleep.time - How much sleep do you usually get at night on weekdays or workdays ? And sleep time was categorized as < 6 hours, 6 - 8 hours and > 8 hours. Education Monthly household income standard included lower income (<= 4 : $0-$1649), mediate income (<= 8: $1650-$4599) and high income (<=12: $4600 and over). Blood lead were analyzed by dividing them into four quintiles according to the range of values.


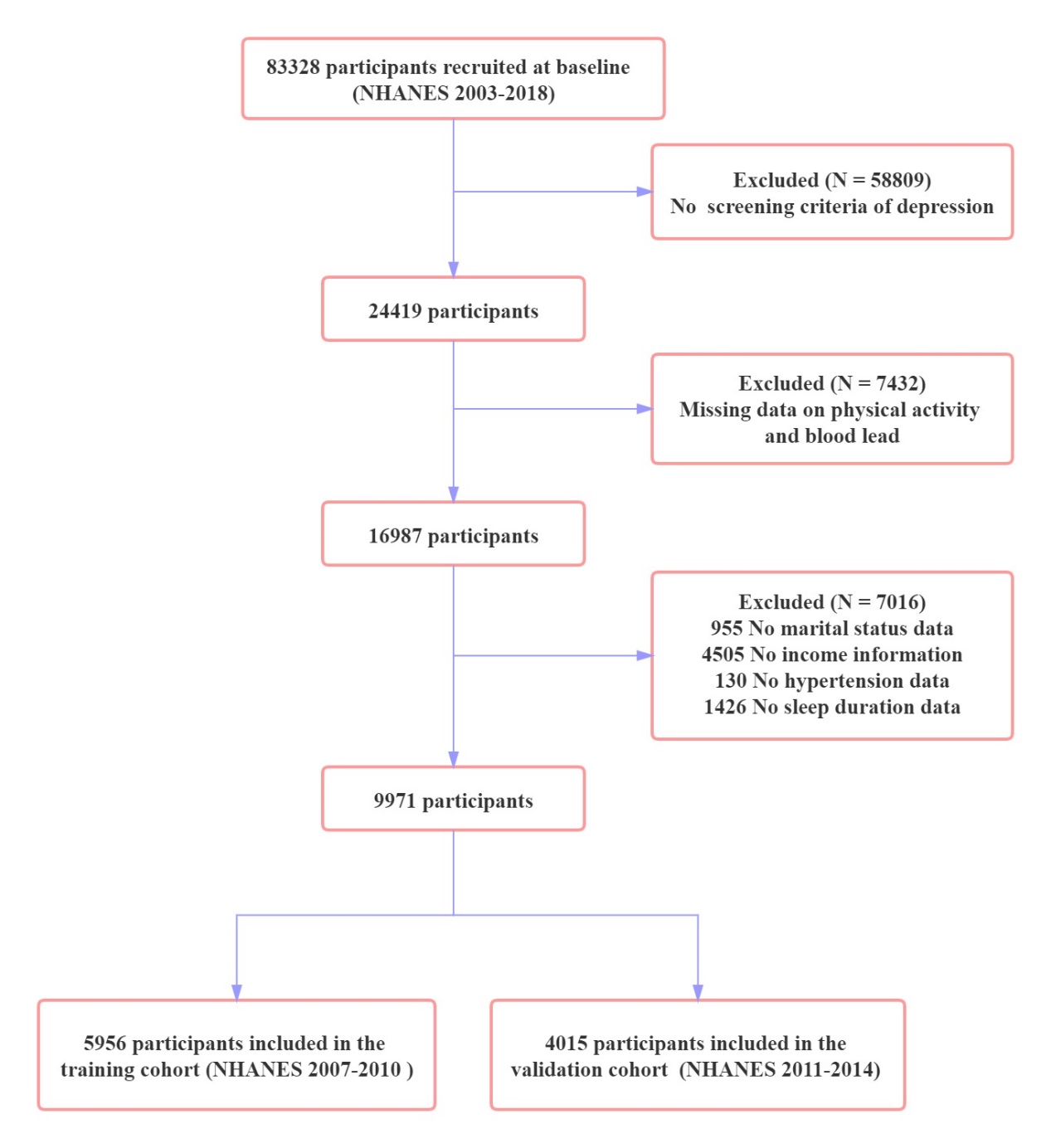


**Figure S1. Samples flow chart.**
